# Dercum's Disease: A Case Report of a Patient Having Both Type 1 and Type 2 Dercum's Disease

**DOI:** 10.1155/2020/6129706

**Published:** 2020-11-10

**Authors:** Cameron Moattari, Richard A. Giovane, Stephanie DiGiovanni Kinsely

**Affiliations:** ^1^State University of New York Downstate Medical Center, Brooklyn, NY 11203, USA; ^2^Regional Medical Center of Central Alabama, Family Medicine, 29L V Stabler Drive, Greenville, AL 36037, USA; ^3^University of Alabama, Department of Family Medicine, 801 Campus Drive, Tuscaloosa, AL 35487, USA

## Abstract

Dercum's disease, or adiposis dolorosa, is a rare disorder which consists of multiple, painful lipomas within the subcutaneous tissue and has a distribution mainly in the abdomen and extremities. Dercum's disease can be defined as in combination with chronic painful adipose tissue. Although the etiology of Dercum's disease is not clear, it is thought to be a combination of a neurological and endocrine disorder. Treatment for this disease is centered at managing pain. Although there is no standard of care for managing pain, there are different pain management regimes that are promising.

## 1. Introduction

Dercum's disease is a rare disorder characterized by chronic pain due to multiple lipomas in the adipose tissue [1]. Epidemiology for the condition is not defined, but the literature suggests a higher prevalence in obese females, aged 35–50 [1].The disease is often accompanied by a number of associated symptoms such as weakness, fatigue, depression, and dementia [2]. The painful adipose tissue can be diffuse and generalized or concentrated in palpable lipomas [3]. The most commonly affected locations are the extremities, the trunk, the pelvic area, and the buttocks [4].The diagnosis of Dercum's disease is a diagnosis of exclusion and is made clinically, although imaging can be helpful. Effective treatment for patients with Dercum's disease is currently lacking as there is no effective long term treatment. The approach to management of Dercum's disease involves pain control through oral medications or direct injections or surgical removal. Surgical interventions, however, such as liposuction and lipectomy have varying outcomes [5, 6], and direct lidocaine injections into the lipomas have been shown to provide only short-term pain relief [7]. Many case reports of treatment with various medications such as corticosteroids and experimental therapies exist in the literature, but large-scale studies are lacking.

## 2. Case Presentation

We present a case of a 48-year-old female who presented to the clinic for established care. The patient had just been discharged from hospice care due to chronic pain secondary to Dercum's disease. Upon chart review, the patient had a medical history of type 2 diabetes, Hashimoto's disease, migraines, and obesity. Regarding her diagnosis of Dercum's disease, a chart review revealed that the patient began having episodic bouts of generalized pain starting at the age of 40. The pain rate was 7–10/10 during her episodes and could last minutes to days, with the longest episode of generalized pain lasting for 32 days. She also began experiencing migraines along with the pain, accompanied by auras as well as blurry vision and photophobia. Her episodes of pain became more frequent, and she began noticing tender lumps around her abdomen. During one episode, she went to the emergency room due to severe pain. Although her medical records could not be obtained, according to her husband, her lab tests were unremarkable; however, a CT scan of her abdomen revealed many small masses in her subcutaneous tissue. Attempts to obtain the CT scan images were not successful as her husband could not recall the specific emergency room she went to. Subsequent biopsies of the masses revealed histopathologic characteristics of lipomas. The patient's clinical picture was in favor of the diagnosis of Dercum's disease. The patient was discharged and was told to follow-up with her primary care doctor, as well as she was given an appointment with an endocrinologist. The patient saw the endocrinologist for further evaluation, and the patient was diagnosed as having both type I and type II Dercum's disease as she initially had widespread pain in her adipose tissue and then developed painful lipomas. She was referred to physical therapy and was managed in an outpatient setting for years for her pain control which included oxycodone 10 mg BID, ibuprofen 800 mg TID, and alprazolam 0.5 mg TID PRN for anxiety. Upon presentation at the clinic, the patient was accompanied by her husband for established care. The patient reported severe constant diffuse pain, poor appetite, and difficulty sleeping because of the pain. A physical exam revealed multiple mobile, tender masses on her neck, arms, chest, and abdomen. The patient was referred to pain management and hospice care for further evaluation and management. Eventually, the patient was lost to follow-up, and means of communication went unanswered

## 3. Discussion

Dercum's disease, or adiposis dolorosa, is a rare disorder consisting of multiple, painful lipomas subcutaneously distributed mainly on the trunk and extremities [1]. Historically, the associated symptoms in Dercum's disease include easy bruisability, sleep disturbances, impared memory, depression, difficulty concentrating, anxiety, diabetes, fatigue, weakness, and joint aches, yet these symptoms are not always present [2]. The minimum definition of Dercum's disease proposed by Hansson et al. is generalized as overweight or obesity in combination with chronic (>3 months) painful adipose tissue [2]. Dercum's disease has been suggested to be classified into 4 distinct variants: type I—the generalized diffuse form presenting with widespread painful adipose tissue with no apparent lipomas; type II—the generalized nodular form presenting with widespread, painful adipose tissue with increased pain surrounding lipomas; type III—the localized nodular form presenting with pain strictly surrounding lipomas; and type IV—the juxta-articular form, presenting as painful adipose tissue near large joints, usually the medial knee [2]. Most cases of Dercum's disease occur sporadically although several reports suggest an autosomal dominant inheritance pattern with a possible genetic heterogeneity favoring women [2]. In the case presented, our patient was diagnosed with having both type I and type II per the endocrinologist in which she saw. It was supported by the fact that she initially had widespread painful tissue which then developed painful lipomas.

Diagnosis of Dercum's disease is based mainly on physical examination after other diagnoses have been ruled out. Such differential diagnoses to consider are fibromyalgia, lipoedema, panniculitis, endocrine disorders, primary psychiatric disorders, multiple symmetric lipomatosis, familial multiple lipomatosis, and adipose tissue tumours. Imaging may aid in diagnosis. MRI may demonstrate multiple small, oblong, fatty lesions in the subcutaneous tissue with a nodular increased fluid signal that appears “blush-like.” Ultrasound may show hyperechoic nodules [4]. There are no clear laboratory markers for the condition. ESR or CPR may be elevated, while autoimmune disease markers are usually negative [2]. One study found that adipose IL-6 and IL-13 levels were significantly elevated in women with Dercum's disease compared to matched controls, while Fractalkine and MIP-1b levels were significantly lower. IL-6, IL-13, Fractalkine, and MIP-1b are glycoproteins and chemokines that are proinflammatory. The clinical significance of these findings is unclear [8]. Regional bioimpedance measures can help in distinguishing lipoedema from Dercum's disease [9].

The etiology of Dercum's disease is not clear. Several theories have been proposed, yet none demonstrates clear evidence. Endocrine and nervous system dysfunction have been suspected but never proven to be implicated. Stretching and pressure on nerves from growing lipomas has been suggested but never shown histopathologically [2]. Lipid metabolism has also been suggested as fatty acid composition determined by gas chromatography/mass spectrometry showed a statistically significantly lower vaccenic acid/stearic acid desaturation index of subcutaneous adipose tissue between participants with Dercum's disease compared with control participants [7]. Inflammation has also been suggested but not been shown to vary significantly on histopathology between fat biopsies of painful and nonpainful areas in patients with Dercum's disease, or compared with obese control patients [10].

Treatment for Dercum's disease revolves around managing pain. Currently, there is no standard of therapy but several options described in numerous case reports exist for reducing symptoms. Lifestyle interventions such as diet and exercise have not been shown to reduce painful fatty tissue. Surgical interventions such as liposuction or lipectomy subjectively improve pain measurements, although this effect diminishes over time. One case report showed reduced pain and increased mobility in 4 patients with the juxta-articular form after dermolipectomy of the medial knee [8]. The utility of surgery in relation to the inherent risks has been called into question, and further research is required [8–10]. Lidocaine injection directly into lipomas has been shown to provide pain relief for up to 4 months [11]. Moreover, intravenous lidocaine showed improvement of pain for up to 9 months [12]. Many medications have been shown to improve pain. NSAIDs were traditionally thought to be ineffective, but a study by Herbst et al. showed reduced pain in 89% of patients with Dercum's disease (*n* = 89) [2, 13, 14]. Corticosteroids given orally or through injection have not been shown to provide any benefit, and one case study showed a link between corticosteroid use and the development of Dercum's disease [15]. In addition, case reports exist supporting the potential use of pregabalin [16], D-amphetamine [16], metformin [17], interferon alfa-2b [18], infliximab, and methotrexate [19] in managing pain. Several novel and noninvasive procedures have also been described as beneficial. Treatment with transcutaneous frequency rhythmic electrical modulation system over 6 months improved pain assessment and reduced subcutaneous adipose tissue in one man with Dercum's disease [20]. A group of 10 patients with Dercum's disease reported improvement in pain after 5 days of rapidly cycling hypobaric pressure [21]. Four weeks of subcutaneous adipose tissue therapy, a form of deep tissue massage, demonstrated a significant decrease in weight but no change in pain scores in several women with Dercum's disease [22]. Randomized, controlled trials are needed to confirm the data described in these studies.

## 4. Conclusion

Dercum's disease is a rare condition that is characterized by painful lipomas and other nonspecific symptoms. Patients with this condition are difficult to diagnose due to its rarity and differing presentations. Managing patients with this condition can prove to be difficult as there is no standard of care for symptom control.

## Data Availability

No data were used to support this study.

## Abbreviations

CT: Computed tomography
IL-6/13: Interleukin-6/13
MIP-1b: Macrophage inflammatory protein-1b
MRI: Magnetic resonance imaging.
